# Trophic Ecology of the Pyjama Shark *Poroderma africanum* (Gmelin, 1789) Elucidated by Stable Isotopes

**DOI:** 10.3390/ani14172559

**Published:** 2024-09-03

**Authors:** Luca Caracausi, Zaira Da Ros, Alice Premici, Enrico Gennari, Emanuela Fanelli

**Affiliations:** 1 Department of Life and Environmental Sciences, Polytechnic University of Marche, Via Brecce Bianche, 60131 Ancona, Italy; caracausi.luca@gmail.com (L.C.); alice.premici@gmail.com (A.P.); e.fanelli@univpm.it (E.F.); 2 Oceans Research Institute, P.O. Box 1767, Mossel Bay 6500, South Africa; e.gennari@oceans-research.com

**Keywords:** stable isotope analysis, catshark, mesopredator, South Africa

## Abstract

**Simple Summary:**

Sharks, as important members of marine food webs, are often targeted by commercial and sport fishing, and moreover they constitute a significant part of the bycatch along with marine mammals and turtles. Overfishing, especially of top-predator species, disrupts the entire food web through a process called “mesopredator release”, where the removal of top predators leads to an increase in mid-level predators, altering the ecosystem balance. Despite their crucial ecological role, there is limited information on sharks’ diets. Traditional methods like stomach content analysis and newer, less invasive techniques, such as stable isotope analysis (SIA) of muscle tissue biopsies, provide insights into their feeding habits. A study on the pyjama shark or striped catshark (*Poroderma africanum*), a species native to South Africa, used SIA to explore its trophic ecology. The study found that as pyjama sharks grow, their diet shifts from more planktonic species as juveniles to more benthic prey. Juvenile sharks have a more varied diet, while adults are more selective. Although the pyjama shark population is increasing, according to the IUCN, there is a lack of structured monitoring programmes and catch data. Understanding the trophic ecology of mesopredators like the pyjama shark is crucial for predicting the impacts of their predation on marine ecosystems.

**Abstract:**

Sharks may occupy both intermediate and upper levels of marine food webs. They are overfished worldwide and constitute one of the largest portions of the bycatch. The removal of top-predator species has negative cascading effects on the entire food web, causing the “mesopredator release” phenomenon, which leads to an increase in mesopredators with consequent changes in the ecosystem’s energetic balance. Despite their important ecological role, information on their trophic ecology is limited. This essential information can be obtained through the analysis of stomach contents and, more recently, by using less invasive techniques, such as the stable isotope analysis of muscle tissue, obtained through biopsies. Here, we analysed the trophic ecology of the pyjama shark or striped catshark *Poroderma africanum*, an endemic species of South Africa, by means of SIA. The results obtained from SIA were analysed using the R SIMMR and SIBER packages to estimate the contribution of potential food sources to the diet and to evaluate the extent of the trophic niches. The SIMMR outputs showed that adults select more benthic prey than juveniles, which consume more planktonic species, with juveniles being more generalist than adults, according to SIBER outputs. As assessed by IUCN, the population of *P. africanum* is increasing, and given its role as mesopredator, future monitoring efforts could be crucial to elucidating their potential effects in marine food webs.

## 1. Introduction

Sharks are cartilaginous fishes that may occupy both intermediate and apical levels of marine food webs. They are often typified as opportunistic predators, with a wide trophic spectrum from plankton to marine mammals [1,2]. Their importance is explained by the likely effects they may have on particular prey species and their high connectivity in the food web [2,3,4]. Sharks have the potential to influence the community structure and the habitat use of prey organisms [5]. They are overfished worldwide as a commercial and sport fishing target, and they constitute one of the largest portions of the bycatch, together with marine mammals and turtles [6]. The removal of top-predator species has negative cascading effects on the entire food web, causing the so-called “mesopredator release” phenomenon, which leads to an increase in mesopredators with a consequent change in the ecosystem’s energetic balance [5,7]. Despite their important ecological role, information on their trophic ecology is limited because of their intrinsic biological characteristics, such as naturally low population densities, generally highly mobile and elusive behaviour and large-distance migrations [8,9]. Information on the composition of their diet is essential and can be obtained through the analysis of stable isotopes (stable isotope analysis or SIA) [10]. SIA reflects assimilated food, but it cannot be used to identify specific prey [11]. Moreover, this method produces information on long-term diet, since the stable isotope values of predators reflect those of assimilated nutrients from ingested prey integrated over longer time periods [12,13]. SIA provides a better understanding of the trophic role of the studied species, which can in turn support appropriate management and conservation strategies [14]. The use of SIA in trophic studies is based on a biological–chemical concept: during normal metabolic functions, the heavier, rare isotope is retained, while the lighter, more common isotope is excreted [15]. The stable isotopes of carbon (*δ*^13^C) and nitrogen (*δ*^15^N) are most used and provide powerful tools for estimating carbon flow to consumers (*δ*^13^C) and related trophic positions of species within food webs (*δ*^15^N). The analysis of nitrogen isotopes provides information on trophic position, as *δ*^15^N increases with increasing trophic level [11,16], while that of carbon isotopes provides insights on the foraging ecology and movement/migration patterns [17]. The type of tissue that will be analysed is recognised as a fundamental aspect of experimental design when applying SIA in ecological studies. Each tissue has its own characteristics, which include different metabolic turnover rates between different species and within the same species. When using muscle tissue, which integrates short- to medium-term information efficiently on assimilated diets [13], a small sample size is required, and it can be obtained through a small biopsy, thus not provoking any damage or stress to the animal. For this project, this approach was used for the pyjama shark or striped catshark, *Poroderma africanum*. This shark belongs to the Scyliorhinidae family and is endemic to the waters off South African coasts, especially Cape Province. It is a nocturnal species, and its main characteristic is the presence of seven distinct dark longitudinal broad stripes extending the entire length of the body, on the dorsal and lateral sides [18]. The species has no specific protection, but its retention in commercial line fisheries is prohibited. The International Union for Conservation of Nature (IUCN) has assessed its population as “Least Concern”.

The main objectives of this study were to investigate the trophic ecology of *P. africanum*. Specifically, we aimed to i. evaluate its feeding habits with SIA; ii. assess any spatial differences in its diet in three sites near Mossel Bay harbour; and iii. discuss trends in its population status in the light of the “mesopredator release hypothesis”.

## 2. Materials and Methods

### 2.1. Study Area and Sampling

**Figure 1:**
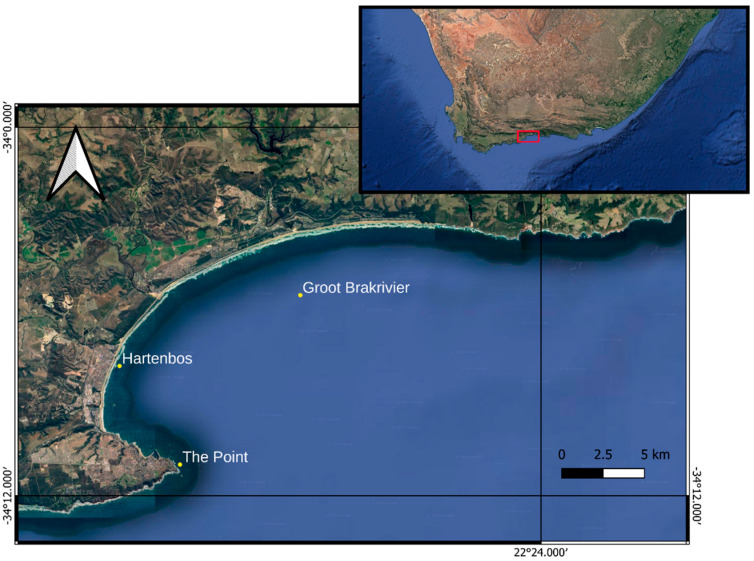
Study area in Mossel Bay, in the Western Cape province of South Africa, with the indication of the sampling sites (yellow symbols) of *P. africanum*. HB = Hartenbos, GB = Groot Brakrivier, TP = The Point (Downloaded on 2 May 2022 and modified from Google Earth Pro).

The study area is in Mossel Bay, in the Western Cape province of South Africa, halfway between Cape Town and Port Elizabeth (each 400 km away). The main feature of this site is the occurrence of a rocky reef. One of the most important abiotic factors in the structure of South African rocky intertidal communities is the role of wave action, which includes implications for energy flow between the intertidal and adjacent ecosystem [19,20,21]. Along the bay, there is an important freshwater contribution deriving from three main rivers, which take their name from the respective cities that pass through: Hartenbos, Little Brakrivier and Groot Brakrivier. All three rivers, crossing agricultural areas and raised beaches, tend to funnel great amounts of nutrients into the sea, enhancing primary production [22]. For this project, three sampling sites were chosen inside the bay. These were, from the closest to the furthest from Mossel Bay harbour, “The Point” (34°10.983′ S 22°09.774′ E, hereafter TP), “Hartenbos” (34°07.775′ S 22°07.392′ E, hereafter HB) and “Groot Brakrivier” (34°05.469′ S 22°14.514′ E, hereafter GB) (Figure 1). 

The collection of muscle samples took place during August 2021. Sharks were captured using a handline or a fishing pole and, once caught and pulled into the boat, they were placed in a container half-filled with salt water to reduce the stress during subsequent processes, like removing the hook from the mouth. Then, their total length (TL in cm), from the tip of the snout to the longest length of the tail, and pre-caudal length (PCL in cm), from the tip of the snout to the pre-caudal dimple, were measured [16]. For sample extraction, a 5 mm biopunch was inserted behind the first dorsal fin and a tiny piece of muscle tissue was taken. Subsequently, the samples were placed in a numbered test tube. At the end, the sharks were immediately released. Finally, all the samples were stored and maintained in a freezer at −20 °C until laboratory analysis.

Collection was authorised by the South African Department of Forestry Fisheries and Environment (RES2019-20) while the samples were exported under the TOPS permit 52015.

### 2.2. Stable Isotope Analysis (SIA)

Sample tissues of *P. africanum* were oven-dried at 60° for 24 h [23]. Each dried sample was grinded using a mortar and a small pestle, and ca. 1–1.2 mg was placed into tin capsules (Elemental Microanalysis Tin Capsules Pressed, Standard Weight 5 × 3.5 mm) using a rectangular tipped spatula. After weighing, each capsule containing the sample was closed using a rectangular-tipped spatula and tweezers and ordered in a plastic rack. Then, the rack was stored in a freezer at −20° until delivery to the specialised laboratory for subsequent analysis. Samples were analysed through an elemental analyser (Thermo Flash EA 1112, Thermo Scientific: Waltham, MA, USA) for the determination of total carbon and nitrogen, coupled with a continuous-flow isotope-ratio mass spectrometer (Thermo Delta Plus XP) for the determination of *δ*^13^C and *δ*^15^N, at the Laboratory of Stable Isotope Ecology of the University of Palermo (Italy). Lipid extraction is important as sharks have a high concentration of lipids because of the presence of large amounts of squalene. This could affect the *δ*^13^C values and thus the results of the analysis. Here, lipids were not extracted from the samples because the number of samples was not sufficient. Thus, a correction equation was applied to *δ*^13^C values, by using the relationship between C:N ratios and the *δ*^13^C signatures according to [24]: *δ*^13^C_corrected_ = *δ*^13^C_untreated_ − 3.32 + 0.99 × C:N_bulk_.

### 2.3. Statistical Analysis

A three-fixed-factor design was used: ‘TL’, ‘Sex’ and ‘Site’. The first two were two-levels factors (‘AD’/‘JUV’ for TL, and ‘Male’/‘Female’ for Sex). The median of the length frequency distribution of *P. africanum* separated juveniles (<82 cm) from adults (≥82 cm). The third factor (Site) has three levels (‘HB’/‘GB’/‘TP’), according to the three sampling sites. Differences in *δ*^15^N and *δ*^13^C contents among the considered factors and their interactions were tested by univariate and multivariate PERMANOVA (Permutational Multivariate Analysis of Variance) and were carried out on the resemblance Euclidean matrix of untransformed *δ*^15^N and *δ*^13^C values, respectively. For all PERMANOVA tests, the significance value was set at *p* < 0.05. Both univariate and multivariate PERMANOVA tests were carried out under an unrestricted permutation of raw data, with 9999 permutations. The Monte Carlo test was applied because of the low sample size. In addition, if the main test of PERMANOVA highlighted significant differences, a pairwise test was performed to identify the source of the variation. Multivariate and univariate statistical analyses on the obtained results were conducted using PRIMER6 and PERMANOVA+ [25,26]. Correlations between *δ*^15^N or *δ*^13^C and the total length of *P. africanum* were calculated with PAST (version 4.0.9) [27].

### 2.4. Mixing Models

**Figure 2:**
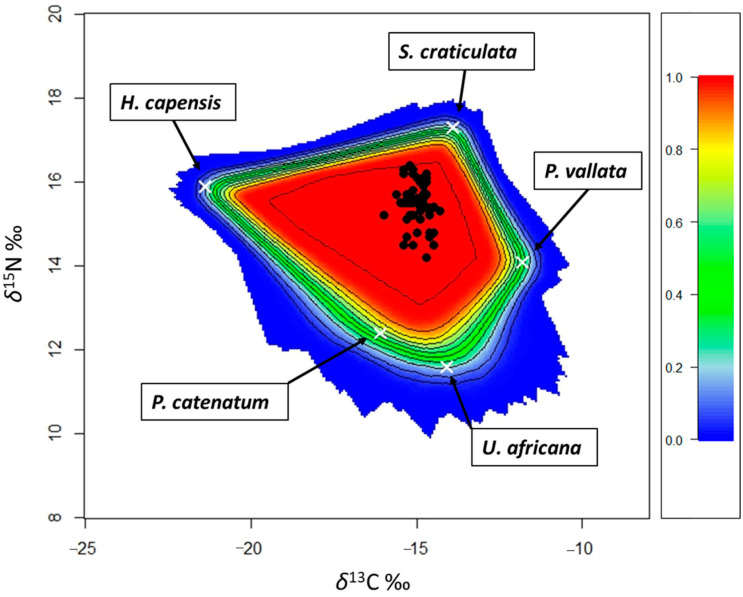
Mixing plot of the potential food sources for *P. africanum*: white crosses indicate mean isotopic values of the food sources, corrected with the TEF. Black dots represent the isotopic values of *P. africanum* samples. Probability contours are at the 5% level (outermost contour) and at every 10% level, following the colors shown in the legend in the right part of the graph. Full name of prey species as in Table 1.

A Bayesian model SIMMR (Stable Isotope Mixing Models in R [28]) was run to estimate the contribution of the different food sources to the diet of *P. africanum* with the software R 4.0.5 (R Development Core Team 2009). The variability in the isotope values (mean and standard deviation) of prey species can be incorporated into the model [29]. Before running the model, the isotopic values of the sources and sharks were plotted, applying the correct trophic enrichment factors (TEFs) to potential sources to build a mixing polygon (Figure 2) [30]. 

TEFs used for *δ*^15^N and *δ*^13^C are 2.29 and 0.9, respectively [31]. The list of potential prey of *P. africanum* was taken from the literature, as there are no data from prey directly taken in South Africa (Table 1).

**Table 1:** List of potential prey of *P. africanum* and the related isotopic composition (plus standard deviations) as reported in the literature.

Sources	Mean *δ*^13^C (‰)	Mean *δ*^15^N (‰)	SD *δ*^13^C (‰)	SD *δ*^15^N (‰)
*Perinereis nuntia vallata* [32]	−12.7	11.8	0.3	0.1
*Sesarma catenata* [32]	−17.0	10.1	1	0.2
*Upogebia africana* [32]	−15.0	9.3	1	0.4
*Hyporhamphus capensis* [33]	−22.3	13.6	0.01	0.01
*Phalium craticulatum* [34]	−14.8	15.0	0.01	0.01

The SIBER package (Stable Isotope Bayesian Ellipses in R 3.5.3) [35] was used to compare isotopic niche widths among and within the communities. It was also used to calculate TA (Total Convex Hull Area) and SEA_C_ (Standard Ellipse Area corrected for low sample size, *p* interval = 0.40 to encompass 40% of our data) [36] for the different communities. Three models were run on *P. africanum* isotopic contents using different combinations. In the first, the three sites were used as groups and a unique community (value 1) was considered. In the second, the three sites were used as groups, but two communities were set, with each one corresponding to one of the two sexes. In the third model, the two levels of TL (juveniles below 81 cm TL and adults >82 cm TL) were used to determine two groups of the same community (Table 2). 

## 3. Results

**Figure 3:**
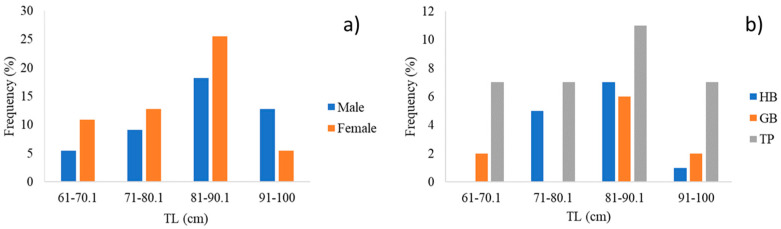
(**a**) Length frequency distribution of males and females of *P*. *africanum* and (**b**) length frequency distribution according to the site (HB = Hartenbos, GB = Groot Brakrivier and TP = The Point) and the total length (TL); *n* = 55.

The length frequency distribution, considering all individuals, shows a different trend for males (*n* = 25) and females (*n* = 30) (Figure 3a). The results indicate that 18% of 25 sampled males of *P*. *africanum* have a TL between 81 and 90.1 cm. Most of the collected specimens have a TL between 91 and 100 cm. Females predominate in the three size classes below 90.1 cm. The results found that 5% have a TL above 91 cm. All size classes are represented at TP, from 61 to 100 cm. At GB, there are no individuals between 71 and 80.1 cm, while at HB, no individuals between 61 and 70.1 cm occurred (Figure 3b).

### 3.1. Overall Isotopic Composition

Isotopic values for all considered levels within factors are shown in Table 3. 

**Figure 4:**
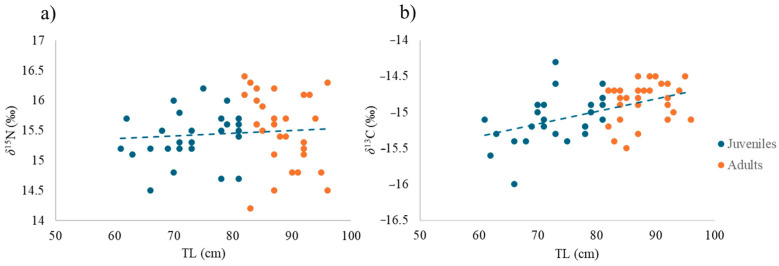
Scatterplot of (**a**) *δ*^15^N (‰) values vs. total length (cm) and (**b**) *δ*^13^C (‰) values vs. total length (cm).

*δ*^13^C values are very similar in all three sites (ca. −15 ± 0.30‰), as are *δ*^15^N values (ca. 15.7 ± 0.30‰). The correlation between TL and *δ*^15^N (R = 0.08, *p* > 0.05, Figure 4a) was positive but not significant, while the correlation of TL with *δ*^13^C (R = 0.5, *p* < 0.001; Figure 4b) was positive and significant. 

The univariate PERMANOVA test carried on *δ*^15^N values (*p* < 0.05) showed significant differences for the factor “Site” (Table 4a). Pairwise tests showed variations between the levels of the factors “Site” (between TP and the other two sites) and “Sex” (between males and females) (Table 4a). The univariate PERMANOVA test on *δ*^13^C values showed significant differences only for factor “TL” (Table 4a).

### 3.2. Mixing Models and Niche Width

**Figure 5:**
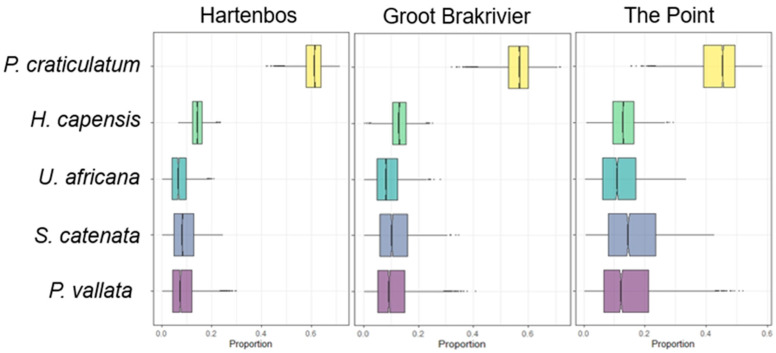
Proportions of each food source in the diet of the catshark collected in Hartenbos (HB), Groot Brakrivier (GB) and The Point (TP). Boxplots were obtained with stable isotope analysis mixing models. Each plot shows proportions for each food source in the specific site. Boxes indicate 50%, 75% and 95% Bayesian confidence intervals.

Two Bayesian SIMMR models were used to estimate the potential food sources for *P*. *africanum*, considering the significance obtained with the univariate analysis. The first concerns the difference between sites: the gastropod *Phalium craticulatum*, in yellow, is the species that contributes the most, about 60 percent, to the diet in all areas, with some differences in the contribution of other species (Figure 5). 

**Figure 6:**
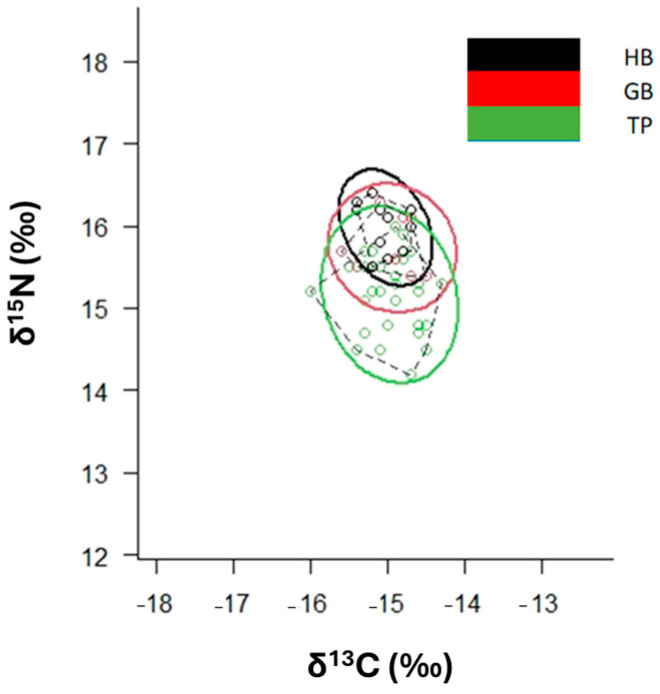
*δ*^13^C-*δ*^15^N scatterplot with standard ellipses (*p* interval = 40%) corrected for small sample size (SEA_C_) for *P. africanum* collected in Mossel Bay, by site (Hartenbos = HB, Groot Brakrivier = GB and The Point = TP).

Standard ellipses obtained by SIBER show the isotopic niche widths of the groups. The niche width of P. *africanum* in TP (area near the harbour) is greater than in the other two sites (Figure 6, Table S1). 

**Figure 7:**
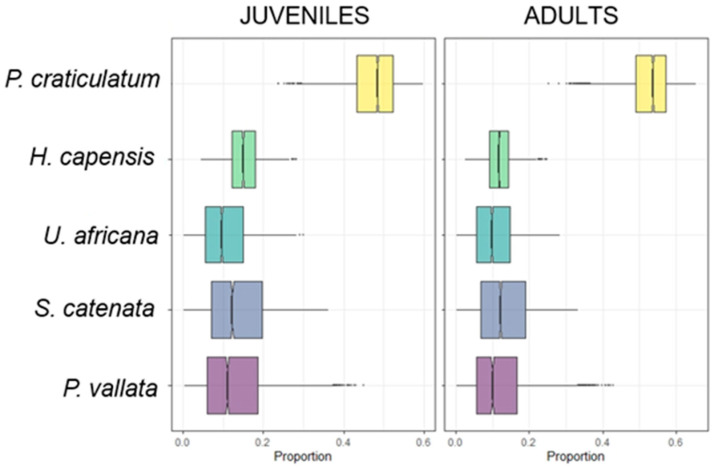
Proportions of each food source in the diet of the catshark collected for juveniles and adults. Boxplots were obtained with stable isotope analysis mixing models. Each plot shows proportions for each food source in the specific TL. Boxes indicate 50%, 75% and 95% Bayesian confidence intervals.

The second, however, concerns the difference between sizes: *Phalium craticulatum* has a greater contribution in adults, while *Hyphoramphus capensis* has a greater contribution in juveniles (Figure 7). 

**Figure 8:**
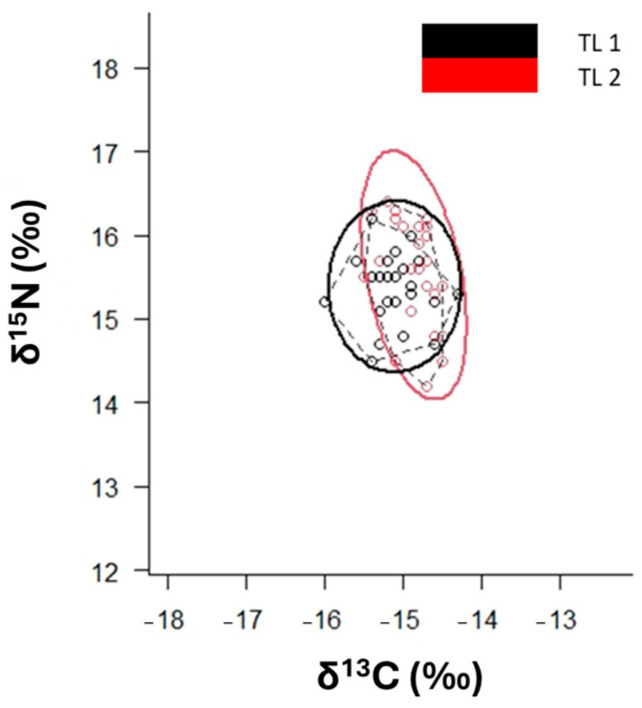
*δ*^13^C-*δ*^15^N scatterplot with standard ellipses (p interval = 40%) corrected for small sample size (SEA_C_) for *P. africanum* collected in Mossel Bay, by total length (TL 1 = juveniles smaller than 81 cm TL, TL 2 = adults larger than 82 cm TL).

The SEA_C_ value of adults is stretched along *δ*^15^N-axis (Figure 8, Table S2). 

## 4. Discussion

**Figure 9:**
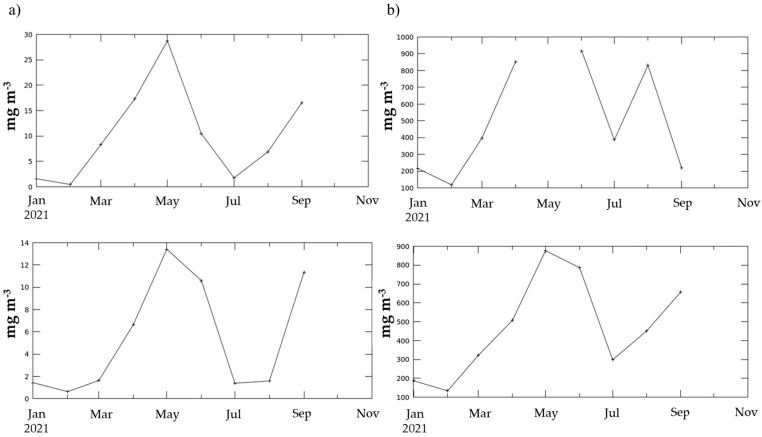
(**a**) Trends of the chlorophyll-a concentration (mg m^−3^) at the three sampling sites (HB and GB considered together, on the top, and TP on the bottom) and (**b**) trends of the particulate organic carbon concentration (mg m^−3^) at the three sampling sites (HB and GB considered together, on the top, and TP on the bottom).

Usually, in catsharks, *δ*^15^N content is greater in animals of a larger size [37,38]. As catsharks grow, they feed on prey positioned at higher trophic levels. They can select specimens of the same species, but of a larger size or located at a higher position within the food web, as also observed in other macropredator sharks, such as smooth-hound sharks [39]. However, this positive relationship between *δ*^15^N and TL is not always valid [37]. In some cases, *δ*^15^N values, and in turn the trophic level, remain fairly constant as the size increases. This happens for the specimens of *Poroderma africanum* analysed here. Conversely, the increasing *δ*^13^C trend with increasing size suggests an ontogenetic dietary shift associated with changes in morphology, physiology or lifestyle [16]. The stable isotope value of carbon indicates whether the carbon source is more planktonic or more benthic [40], or marine vs. continental, thus providing indications of an inshore and offshore trend [41,42]. Higher *δ*^13^C values (from −13 to −17‰) indicate that a species is relying on a benthic food web. Conversely, lower *δ*^13^C values point to a greater dependence on a pelagic food web [40,41]. In this study, juveniles showed very low *δ*^13^C values, while adults had a higher *δ*^13^C value, with a significant increase in *δ*^13^C with catsharks’ size. Two hypotheses can be advanced for this finding: (i) juveniles eat more benthopelagic shrimps or other prey approaching the sea bottom, while adults feed on purely benthic items [43]; (ii) the shift in the isotopic signal could be due to a horizontal displacement with adults living more offshore than juveniles [44,45]. However, this catshark is not recognised as a migrator, so it is unlikely that there is an inshore–offshore movement for this species [46], particularly in this study area. Moreover, the output of the SIBER model carried out considering TL as a discriminant factor shows a higher selective diet of adults (that show a stretched SEA_C_ along the *δ*^15^N-axis) compared to the more generalist diet of juveniles. This is likely because most of the adults analysed in this study live near The Point (TP), which, probably due to input from organic discharges, has the most variable source of N among the three sampling sites. The TP site is close to the harbour, while the other two sites are characterised by the presence of two rivers. The output of the second SIMMR model, which considers the differences between juveniles and adults, confirms that the mean contribution of the gastropod *Phalium craticulatum* (a strictly benthic organism) to adults’ diet is greater than for juveniles. Similarly, the pelagic fish *Hyporhamphus capensis* seems to give a greater contribution to juveniles’ diet. However, since the isotopic data used on the model for prey were from the literature, the model results must be taken with caution and further studies are needed on *P. africanum’s* diet to compare SIA to SCA results. Additionally, some environmental variables (downloaded at https://giovanni.gsfc.nasa.gov/giovanni/, accessed on 22 March 2024) may explain the observed pattern: river runoff, chlorophyll-a concentration, as a proxy of primary production, and particulate organic carbon (POC) concentration. Satellite data (MODIS-Aqua, Modisa, Level 3m) confirmed the difference in chlorophyll-a and POC concentrations among the three sites (data up 30th October 2021) (Figure 9a,b). 

Higher values of these two variables were recorded at HB and GB than at TP. No differences were registered among the runoff values in the three sites, in agreement with the similar *δ*^13^C values observed in the three sites [47,48].

Although *Poroderma africanum* has an important ecological role in the local food web, as also highlighted by our results, catch data are scarce. This implies the lack of a well-defined management plan for the species that allows its adequate conservation or, at least, adequate monitoring of its population status in South African waters, as also reported by the IUCN assessment for this species.

## 5. Conclusions

The scyliorhinid *P. africanum* acts as a mesopredator, occupying the intermediate level of the food web it belongs to. If mesopredators increase, they could lead other species, which share the same resources, such as benthic prey, to modify their trophic niche and possibly also their habitats [5,49], thus avoiding competitive exclusion. Furthermore, an increase in mesopredators would even have the potential to cause the extinction of some prey, particularly the more sensitive species, who have low population growth rates or are more easily attacked by mesopredators [5]. Future studies, for example those conducted with stomach content analyses by recovering samples from bycatch, could confirm which are the specific prey ingested by *P. africanum,* shedding more light on its feeding ecology and trophic position, together with its ecological role.

## Figures and Tables

**Table 2:** Groups and communities considered for running SIBER on SIA results.

Model	Groups	Communities
1	TP, HB and GB	1
2	TP, HB and GB	2 (M and F)
3	AD and JUV	1

**Table 3:** *δ*^13^C and *δ*^15^N (mean and standard deviation = SD) for F (females), M (males), JUV (juveniles), AD (adults), HB (Hartenbos), GB (Groot Brakrivier) and TP (The Point).

	Mean *δ*^13^C (‰)	SD *δ*^13^C (‰)	Mean *δ*^15^N (‰)	SD *δ*^15^N (‰)
F	−15	0.3	15.5	0.4
M	−14.9	0.3	15.4	0.6
JUV	−15.1	0.3	15.4	0.4
AD	−14.9	0.3	15.5	0.6
HB	−15.0	0.2	16.0	0.3
GB	−14.9	0.3	15.7	0.3
TP	−15.0	0.4	15.2	0.4

**Table 4:** Results of the PERMANOVA main test (**a**) and of the pairwise comparisons (**b**) for *δ*^15^N (left) and *δ*^13^C (right). df = degrees of freedom; MS = mean square; Pseudo-F = statistic F; t = statistic t for pairwise comparisons; p(MC) = probability level with Monte Carlo test; TL = total length; HB = Hartenbos; GB = Groot Brakrivier; TP = The Point; F = female; M = male; * = *p* < 0.05; n.s. = not significant.

(**a**)		***δ*^15^N**			***δ*^13^C**		
**Source**	**df**	**MS**	**Pseudo-F**	**P(MC)**	**MS**	**Pseudo-F**	**P(MC)**
TL	1	0.22	1.60	n.s.	0.71	7.12	*
Sex	1	0.00	0.03	n.s.	0.00	0.04	n.s.
Site	2	3.18	23.38	*	0.04	0.35	n.s.
TL×Sex	1	0.00	0.00	n.s.	0.01	0.11	n.s.
TL×Site	2	0.19	1.39	n.s.	0.11	1.15	n.s.
Sex×Site	2	0.40	2.95	n.s.	0.14	1.46	n.s.
TL×Sex×Site	2	0.19	1.38	n.s.	0.06	0.57	n.s.
Residuals	43	0.14			0.10		
Total	54						
(**b**)							
**Groups**	**t**	**P(MC)**					
HB, GB	0.94	n.s.					
HB, TP	4.94	*					
GB, TP	4.16	*					
**Groups**	**t**	**P(MC)**					
F, M	2.19	*					

## Data Availability

Data are made available upon request to the authors.

## Supplementary Materials

The following supporting information can be downloaded at https://www.mdpi.com/article/10.3390/ani14172559/s1: Table S1: Total Convex Hull Area (TA) and Standard Ellipse Areas corrected for small sample size (SEA_C_) values of *P. africanum* between sites. Table S2: Total Convex Hull Area (TA) and Standard Ellipse Areas corrected for small sample size (SEA_C_) values of *P. africanum* between TLs.
