# Supplementary material for: Trophic Ecology of the Pyjama Shark Poroderma africanum (Gmelin, 1789) Elucidated by Stable Isotopes
Source: Animals (Basel). 2024 Sep 3;14(17):2559. doi: 10.3390/ani14172559 (PMC11394248; doi:10.3390/ani14172559)
Supplement: Supplementary file 1 [file animals-14-02559-s001.zip › animals-3160841-supplementary.pdf]

animals

Article

# Trophic Ecology of the Pyjama Shark *Poroderma africanum* (Gmelin, 1789) Elucidated by Stable Isotopes

**Luca Caracausi [1], Zaira Da Ros [1,*], Alice Premici [1], Enrico Gennari [2] and Emanuela Fanelli [1]**

[1] Department of Life and Environmental Sciences, Polytechnic University of Marche, Via Brecce Bianche, 60131 Ancona, Italy; caracausi.luca@gmail.com (L.C.); alice.premici@gmail.com (A.P.); e.fanelli@univpm.it (E.F.)
[2] Oceans Research Institute, P.O. Box 1767, Mossel Bay 6500, South Africa; e.gennari@oceans-research.com
* Correspondence: z.daros@staff.univpm.it

## Supplementary Materials

**Table S1.** Total Convex Hull Area (TA) and Standard Ellipse Areas corrected for small sample size (SEAc) values of *P. africanum* between sites. Results are expressed as ‰$^2$.

| | HB | GB | TP |
|---|---|---|---|
| **TA** | 0.45 | 0.62 | 1.75 |
| **SEAc** | 0.23 | 0.38 | 0.51 |

**Table S2.** Total Convex Hull Area (TA) and Standard Ellipse Areas corrected for small sample size (SEAc) values of *P. africanum* between TLs. Results are expressed as ‰$^2$.

| | Adult | Juveniles |
|---|---|---|
| **TA** | 1.56 | 1.77 |
| **SEAc** | 0.50 | 0.47 |
